# Prostate Cancer Proliferation Is Affected by the Subcellular Localization of MCT2 and Accompanied by Significant Peroxisomal Alterations

**DOI:** 10.3390/cancers12113152

**Published:** 2020-10-27

**Authors:** Isabel Valença, Ana Rita Ferreira, Marcelo Correia, Sandra Kühl, Carlo van Roermund, Hans R. Waterham, Valdemar Máximo, Markus Islinger, Daniela Ribeiro

**Affiliations:** 1 Institute of Biomedicine (iBiMED), Department of Medical Sciences, University of Aveiro, 3810-193 Aveiro, Portugal; isabelvalenca@gmail.com (I.V.); arferreira@ua.pt (A.R.F.); 2 i3S-Instituto de Investigação e Inovação em Saúde, University of Porto, 4200-135 Porto, Portugal; mcorreia@ipatimup.pt (M.C.); vmaximo@ipatimup.pt (V.M.); 3 IPATIMUP-Institute of Molecular Pathology and Immunology, University of Porto, 4200-135 Porto, Portugal; 4 Neuroanatomy, Medical Faculty Mannheim, University of Heidelberg, 68167 Mannheim, Germany; sandra.kuehl@medma.uni-heidelberg.de (S.K.); markus.islinger@medma.uni-heidelberg.de (M.I.); 5 Laboratory Genetic Metabolic Diseases, Department of Clinical Chemistry, Amsterdam UMC—Location AMC, 1105 AZ Amsterdam, The Netherlands; c.vanroermund@amsterdamumc.nl (C.v.R.); h.r.waterham@amsterdamumc.nl (H.R.W.); 6 Department of Pathology, Medical Faculty, University of Porto, 4200-319 Porto, Portugal

**Keywords:** peroxisomes, MCT2, prostate cancer

## Abstract

**Simple Summary:**

Fatty acid β-oxidation is a dominant bioenergetic pathway in prostate cancer. It has recently been suggested that the specific targeting of monocarboxylate transporter 2 (MCT2) to peroxisomes contributed to an increase in β-oxidation rates and maintenance of the redox balance in prostate cancer cells. Here we provide evidence demonstrating that prostate cancer streamlines peroxisome metabolism by upregulating distinct pathways involved in lipid metabolism. Importantly, we show that the localization of MCT2 at peroxisomes is required for prostate cancer cell proliferation. Our results emphasize the importance of peroxisomes for prostate cancer development and highlight different cellular mechanisms that may be further explored as possible targets for prostate cancer therapy.

**Abstract:**

Reprogramming of lipid metabolism directly contributes to malignant transformation and progression. The increased uptake of circulating lipids, the transfer of fatty acids from stromal adipocytes to cancer cells, the *de novo* fatty acid synthesis, and the fatty acid oxidation support the central role of lipids in many cancers, including prostate cancer (PCa). Fatty acid β-oxidation is the dominant bioenergetic pathway in PCa and recent evidence suggests that PCa takes advantage of the peroxisome transport machinery to target monocarboxylate transporter 2 (MCT2) to peroxisomes in order to increase β-oxidation rates and maintain the redox balance. Here we show evidence suggesting that PCa streamlines peroxisome metabolism by upregulating distinct pathways involved in lipid metabolism. Moreover, we show that MCT2 is required for PCa cell proliferation and, importantly, that its specific localization at the peroxisomal membranes is essential for this role. Our results highlight the importance of peroxisomes in PCa development and uncover different cellular mechanisms that may be further explored as possible targets for PCa therapy.

## 1. Introduction

Malignant transformation requires multiple metabolic adaptations to respond to energy requirements and to support high cell proliferation rates [1]. Initial studies reported an increase of glucose uptake and glycolysis in cancer cells, even when oxygen was not limiting, leading to an increased lactate production (Warburg effect) [2]. Despite most studies being focused on glycolysis and glutaminolysis, recent evidence suggests that the reprogramming of cellular lipid metabolism contributes directly to malignant transformation and progression [3,4]. Several cancers induce the *de novo* lipid synthesis, increasing e.g., the production of new phospholipids to build new cell membranes, the production of signaling molecules, the escape from oxidative stress, and the acquisition of drug resistance [5,6]. Besides increased lipogenesis, some cancers also take advantage from fatty acid oxidation for energy production [4,7].

Prostate cancer (PCa) has an exclusive metabolic profile: PCa cells exhibit lower glucose consumption rates than most tumor cells and lipids become the main energy source [8]. The central role of lipids in PCa malignancy is supported by the increased uptake of circulating lipids, transfer of fatty acids from stromal adipocytes to PCa cells, and the *de novo* fatty acid synthesis and fatty acid oxidation [9,10,11]. PCa is also conditioned by the response to androgens, which significantly modulate *de novo* fatty acid synthesis [12].

Although most studies have been focused on *de novo* lipid synthesis, recent data highlights the role of increased fatty acid β-oxidation, which appears to be the dominant bioenergetic pathway in PCa [13,14]. It was also shown that peroxisomal branched-chain fatty acid β-oxidation is induced in PCa [13,15,16]. In this context, alpha-methylacyl-CoA racemase (AMACR) was shown to be overexpressed in PCa tissues compared to normal prostate tissues [15,16,17], leading to its recognition as a PCa biomarker [16]. Subsequently, we and others have shown that not only AMACR, but also key enzymes involved in peroxisomal branched-chain β-oxidation, including acyl-CoA oxidase 3 (ACOX3) and D- bifunctional protein (DBP), were upregulated in PCa [13,18].

We have previously reported that the monocarboxylate transporter 2 (MCT2), usually associated with glucose metabolism and overexpressed in cancer [19,20], localizes mainly to peroxisomes in PCa cells derived from localized tumors [18]. PCa tumor cells seem to take advantage of the peroxisomal protein transport machinery in order to target MCT2 to this organelle via peroxin 19 (PEX19), potentially ensuring higher rates of β-oxidation and contributing to the maintenance of the redox balance [18,21]. Although MCTs are commonly associated with glucose metabolism, MCT2 seems to have a crucial role in malignant transformation of prostate cells. Its expression is more evident in prostatic intraepithelial neoplasia (PIN) lesions and localized prostate tumors, compared to nontumoral tissues and metastasis [18,19]. Interestingly, a clear change in peroxisome morphology across prostate malignant transformation was observed, which correlated with MCT2’s presence at this organelle [18]. These results provided evidence for the involvement of peroxisomes and MCT2 in the process of prostate tumor initiation.

In this study, we aimed to further unravel the importance of peroxisome metabolism and morphology as well as the role of MCT2 in PCa. We observed the upregulation of several processes involved in peroxisome lipid metabolism and furthermore demonstrate that the presence of MCT2 at peroxisomes is required for PCa proliferation.

## 2. Results

### 2.1. Peroxisome Metabolism Is Significantly Altered in PCa Cells

**Figure 1:**
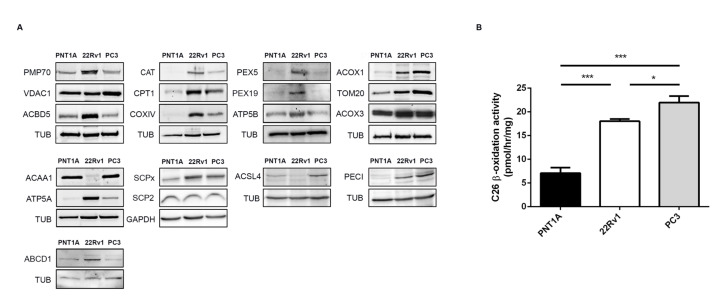
Peroxisome and mitochondria protein and metabolic changes in prostate cancer (PCa). (**A**) Western blot analysis showing the expression levels of the peroxisomal proteins 70-kDa peroxisomal membrane protein (PMP70), acyl-CoA-binding domain-containing protein 5 (ACBD5), catalase (CAT), peroxin 5 (PEX5), peroxin 19 (PEX19), acyl-CoA oxidase 1 (ACOX1), acyl-CoA oxidase 3 (ACOX3), 3-ketoacyl-CoA thiolase (ACAA1), sterol carrier protein x (SCPx), acyl-CoA synthetase 4 (ACSL4), 3,2-trans-enoyl-CoA isomerase (PECI), and ATP binding cassette subfamily D member 1 (ABCD1), and the mitochondrial proteins voltage-dependent anion-selective channel protein 1 (VDAC1), carnitine palmitoyl transferase 1 (CPT1), cytochrome c oxidase subunit 4 (COXIV), ATP synthase subunit alpha and beta (ATP5A, ATP5B), mitochondrial import receptor subunit (TOM20), and sterol carrier protein 2 (SCP2), representative of at least three independent experiments. Tubulin (TUB) and glyceraldehyde 3-phosphate dehydrogenase (GAPDH) were used as loading controls. A densitometric quantification of the immunoblots is presented in Figure S1. (**B**) Cerotic acid (C26:0) peroxisomal β-oxidation activity in PNT1A, 22Rv1, and PC3 cells. Data represent means of three independent experiments and the bars represent standard error of the mean (SEM) of the mean. * *p* < 0.05 *** *p* < 0.001.

It has previously been demonstrated that PCa induces peroxisomal branched-chain fatty acid β-oxidation, increasing the expression of key proteins involved in this pathway [13,18]. In order to further identify peroxisome-dependent pathways that may be involved in PCa malignant transformation and progression, we analyzed the expression of a set of peroxisomal proteins in different cellular models representing distinct stages of PCa disease progression: PNT1A cells (nontumor immortalized adult prostatic epithelial cells), 22Rv1 cells (derived from localized prostate tumor), and PC3 cells (derived from prostate cancer bone metastasis) (Figure 1 and Figure S1).

Our data shows an increase in the expression of most of the tested peroxisomal proteins in 22Rv1 cells, when compared to PNT1A, suggesting important peroxisome-related metabolic alterations between these two cell types. In general, peroxisomal fatty acid degradation pathways seem to be upregulated in 22Rv1 cells, relatively to nontumor cells. This is reflected by a higher potential in fatty acid uptake, suggested by the augmented expression of the 70-kDa peroxisomal membrane protein (PMP70, also known as ABCD3, ATP binding cassette subfamily D member 3) and the ATP binding cassette subfamily D member 1 (ABCD1). While PMP70 preferentially imports hydrophilic branched-chain, polyunsaturated fatty acids and dicarboxylic acids [22,23], but also straight long-chain fatty acids [24], ABCD1 is mainly responsible for the import of very long-chain fatty acids [23]. The higher expression of acyl-CoA-binding domain-containing protein 5 (ACBD5) may not only reflect an increase in very long-chain fatty acid import [25] but may also reveal alterations in contact sites between peroxisomes and the endoplasmic reticulum (ER) [26,27] in PCa cells. Elevated levels of acyl-CoA oxidase 1 (ACOX1), ACOX3, and peroxisomal 3,2-trans-enoyl-CoA isomerase (PECI) further suggest an increased peroxisomal β-oxidation of straight-chain fatty acids (via ACOX1), branched-chain fatty acids (via ACOX3), and unsaturated fatty acids (via PECI). In line with an increase of peroxisomal H_2_O_2_ produced by the ACOXs, catalase (CAT) was found to be also upregulated. The import of matrix and membrane peroxisomal proteins (via PEX5 and PEX19, respectively) appears to be also increased in these cells (Figure 1A).

Remarkably, the expression levels of PMP70, ACBD5, CAT, PEX5, ABCD1, and PEX19 decrease in PC3 cells when compared with 22Rv1 cells (Figure 1A). In contrast, the expression of ACOX1 and PECI increases in PC3 cells. Moreover, the expression of 3-ketoacyl-CoA thiolase (ACAA1), responsible for the final thiolytic cleavage in peroxisomal β-oxidation, and acyl-CoA synthetase 4 (ACSL4) involved in the activation of polyunsaturated long-chain fatty acids to their correspondent acyl-CoA [28] are significantly decreased in 22Rv1 cells, but recovered in PC3 cells. The expression of sterol carrier protein X (SCPx), which is obligatory for the peroxisomal thiolytic breakdown of branched-chain fatty acids and bile acid intermediates [29], is clearly increased in both tumor cells, suggesting, along with the increases in ACOX3 and AMACR [18], that branched-chain fatty acids metabolism may play an important role in PCa cells.

These immunoblot results indicate that peroxisomal β-oxidation is increased in PCa cells. In order to confirm this assumption, we measured the β-oxidation rates of cerotic acid (C26:0) in the three cells lines. Indeed, both 22Rv1 and PC3 cells exhibit significantly higher peroxisomal β-oxidation activities than the nontumor PNT1A cells (Figure 1B). Interestingly, cerotic acid β-oxidation activities are highest in PC3 cells, in line with the higher protein abundance of enzymes representing straight-chain fatty acid catabolism (ACOX1 and ACAA1) [30], and somewhat lower in 22Rv1 cells, which exhibit a more prominent induction of proteins involved in branched-chain fatty acid catabolism (ACOX3, SCPx, PMP70).

As peroxisomes and mitochondria cooperate in many metabolic pathways, we additionally analyzed the expression of several mitochondrial proteins in the same cell models. Our results showed that the expression of carnitine palmitoyl transferase 1 (CPT1), cytochrome c oxidase subunit 4 (COXIV), ATP synthase subunit alpha and beta (ATP5A and B), and mitochondrial import receptor subunit (TOM20) is increased in 22Rv1 cells, suggesting that PCa increases the import of mitochondrial proteins (via TOM20), mitochondrial acyl-CoA transport (via CPT1), and ATP production (via COXIV, ATP5A, and ATP5B) (Figure 1A). The expression of these proteins (with the exception of TOM20) is decreased in PC3 cells, where an increase in the expression of the voltage-dependent anion-selective channel protein 1 (VDAC1) is observed. Of note, it was recently published that PC3 cells rely predominantly on glycolytic energy production, whereas the mitochondrial electron transport was largely nonfunctional [31]. Interestingly, there are no differences in the expression levels of sterol carrier protein 2 (SCP2) which represents, in addition to the peroxisomal, also the nonperoxisomal proportion of SCP2 localizing in the cytosol, ER, and potentially mitochondria [32]. Since peroxisomes, and therefore peroxisomal SCP2, constitute only a minor proportion of the total cellular protein [33], these differences in SCPx/SCP2 expression underline the distinct metabolic changes occurring at peroxisomes of PCa cells.

Our results suggest key changes in peroxisome metabolism in PCa and highlight the role of this organelle in the PCa metabolic transformation.

### 2.2. The Presence of MCT2 at the Peroxisomal Membranes Affects the Organelle’s Morphology

**Figure 2:**
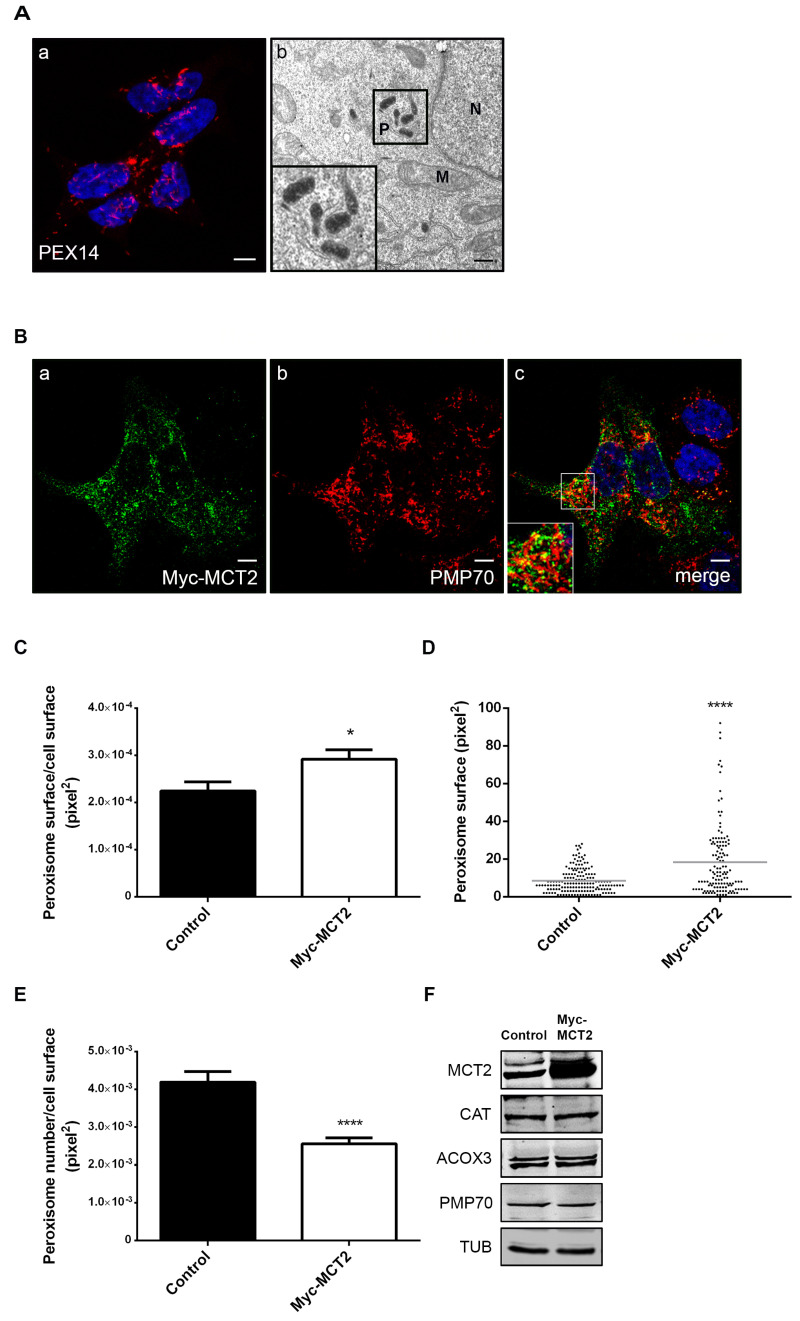
MCT2 overexpression increases peroxisome surface, decreases peroxisome number, and does not affect the expression of key proteins involved in β-oxidation in 22Rv1 PCa cells: (**A**) peroxisomes form clusters in 22Rv1 cells, as shown by (**a**) immunofluorescence analysis of PEX14 by confocal microscopy and (**b**) DAB-staining based transmission electron microscopy; (P) peroxisomes, (M) mitochondria, and (N) nucleus. Nuclei are shown in blue (stained with Hoechst 33258) in (**a**). White bar represents 5 μm and black bar represents 0.5 μm. (**B**–**E**) Analysis of modifications in peroxisome morphology and number in 22Rv1 cells transfected with Myc-MCT2, when compared with nontransfected cells. (**B**) Immunofluorescence analysis of (**a**) Myc and (**b**) PMP70 by confocal microscopy in Myc-MCT2-transfected 22Rv1 cells; (**c**) merge image of a and b, presenting nuclei in blue. (**C**) Quantification analysis of alterations in peroxisomes’ area (pixel^2^), presented as the mean of peroxisome area per total cell area. (**D**) An example of a representative single-cell analysis is presented as the mean of peroxisome area in a cell. (**E**) Quantification analysis of changes in peroxisomes’ number, presented as the mean of peroxisome number per cell area (pixel^2^). Data represent means of three independent experiments and the bars represent SEM of the mean. * *p* < 0.05 **** *p* < 0.0001. (**F**) Western blot analysis showing the expression levels of MCT2, CAT, ACOX3, PMP70, and TUB in control and Myc-MCT2-transfected 22Rv1 cells. A densitometric quantification of the immunoblots is presented in Figure S1.

We have previously shown that peroxisome morphology significantly changes across prostate malignant transformation: when compared to nontumor prostate cells (such as PNT1A), 22Rv1’s peroxisomes show an unusual morphology, appearing slightly elongated and in clusters [18]. Here we used electron microscopy to further analyze peroxisome morphology in 22Rv1 cells and our results indicate that the peroxisomal aggregates previously observed by confocal microscopy constitute local assemblies of elongated peroxisomes (Figure 2A).

The peroxisomal metabolic and morphological changes between nontumor and localized tumor cells have been found to be correlated with the presence of MCT2 at this organelle’s membranes [18]. In order to further analyze the influence of MCT2 on peroxisome morphology and metabolism we assessed the effect of its overexpression and silencing in 22Rv1 cells.

To analyze the effect of MCT2’s overexpression, 22Rv1 cells were transfected with a Myc-MCT2 construct, which, such as the endogenous protein, was preferentially targeted to peroxisomes, as shown by its colocalization with the peroxisomal protein PMP70 (Pearson correlation coefficient of r = 0.61) (Figure 2B). Interestingly, the presence of this overexpressed protein at peroxisomes led to an increase of the peroxisomes’ cellular area compared to nontransfected cells (Figure 2C). Figure 2D shows data from one representative transfected single cell, where an increase in the number of bigger peroxisomes can be observed when compared to a control cell. Furthermore, we also observed a decrease in peroxisome number in transfected cells in response to Myc-MCT2 overexpression (Figure 2E). Although 22Rv1 cells already present an unusual peroxisome morphology, the increased amount of MCT2 at the organelle’s membranes seemed to induce further morphological changes. These results hence suggest a correlation between MCT2’s presence at the peroxisomal membranes and the organelle’s unusual morphology in PCa localized tumor cells. We repeated the analysis in PC3 cells (Figure S2) and observed that, such as the endogenous protein, Myc-MCT2 presented a lower colocalization level with PMP70 (Pearson correlation coefficient of r = 0.40) when compared to 22Rv1 cells. Our results in PC3 cells revealed no relevant differences in the peroxisomal cellular area and only a slight decrease in peroxisome number upon Myc-MCT2 overexpression. We also overexpressed Myc-MCT2 in PNT1A cells and observed no colocalization with the peroxisomal marker, highlighting the specificity of this organelle targeting in prostate tumor cells.

We have previously suggested a putative role for peroxisomes in malignant transformation, associated with the presence of MCT2 at its membranes and proposed that this might be correlated with an increase in peroxisomal β-oxidation levels [18].We aimed to assess whether a further increase in the amount of MCT2 at peroxisomes in 22Rv1 cells (induced by overexpression, as before) would have any effect on the expression of key proteins involved in peroxisomal β-oxidation. Our results have, however, shown that MCT2 overexpression did not further affect the expression of CAT, ACOX3, or PMP70 in these cells (Figure 2F).

**Figure 3:**
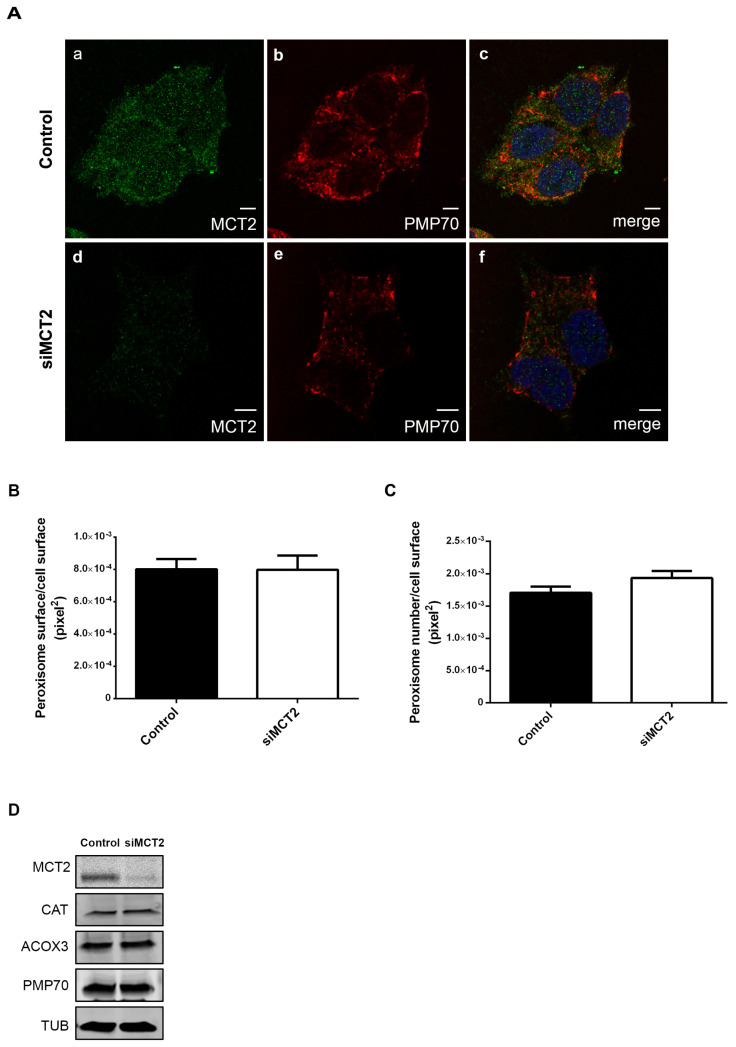
MCT2 knockdown does not interfere with peroxisome dynamics or the expression of key proteins involved in β-oxidation in 22Rv1 PCa cells: (**A**–**C**) analysis of modifications in peroxisome morphology and number in 22Rv1 cells upon silencing of MCT2, when compared with nonsilenced cells. (**A**) Immunofluorescence analysis of (**a**,**d**) MCT2 and (**b**,**e**) PMP70 by confocal microscopy in 22Rv1 cells; (**c**,**f**) merge image of (**a**,**b**) and (**d**,**e**), respectively, presenting nuclei in blue. Bars represent 5 μm. (**B**) Quantification analysis of alterations in peroxisomes’ area (pixel^2^), presented as the mean of peroxisome area per total cell area. (**C**) Quantification analysis of changes in peroxisomes’ number, presented as the mean of peroxisome number per cell area (pixel^2^). Data represent means of three independent experiments and the bars represent SEM of the mean. (**D**) Western blot analysis showing the expression levels of MCT2, CAT, ACOX3, PMP70, and TUB in control and MCT2-silenced 22Rv1 cells. A densitometric quantification of the immunoblots is presented in Figure S1.

As previously mentioned, we also analyzed peroxisome number and morphology upon MCT2 knockdown by RNAi in 22Rv1 cells. Our results show no significant alterations on peroxisome morphology or number (Figure 3A–C), nor on the levels of the peroxisomal membrane protein PMP70 (Figure 3D). These results indicate that the unusual peroxisome morphology, typical for 22Rv1 cells, was not reversed by a transient decrease of MCT2. We also analyzed the effect of MCT2 knockdown in the expression of CAT and ACOX3, which resulted in no considerable changes (Figure 3D). These analyses were also performed in PC3 cells (Figure S3) and, similarly, no significant changes in peroxisome morphology were observed.

### 2.3. MCT2 Localization at the Peroxisomal Membranes Is Associated with PCa Proliferation

**Figure 4:**
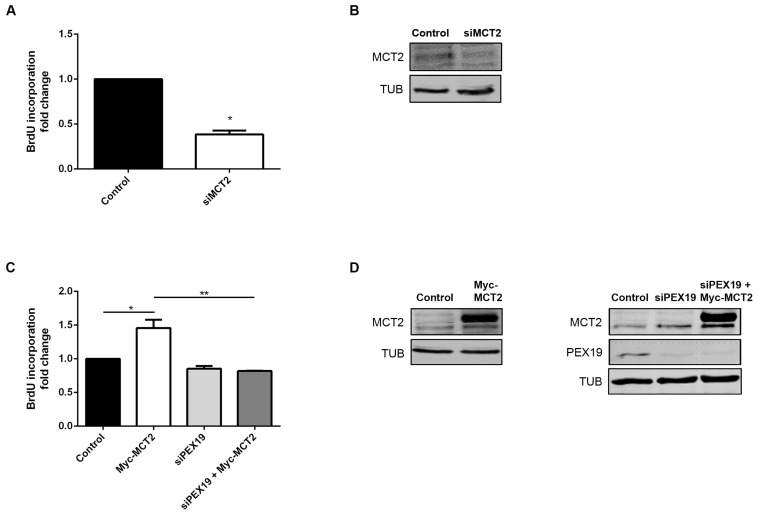
MCT2 localization at the peroxisomal membranes is associated with PCa proliferation. (**A**) Effect of the silencing of MCT2 on 22Rv1 cell proliferation, measured by BrdU incorporation assay. Values are presented in fold change compared to control cells. Data represent means of three independent experiments and the bars represent SEM of the mean. * *p* < 0.05 ** *p* < 0.01 (**B**) Western blot analysis showing the expression levels of MCT2 and TUB in control and MCT2-silenced 22Rv1 cells. (**C**) Effect of Myc-MCT2 overexpression, silencing of Pex19, and MCT2 overexpression in the absence of PEX19 on 22Rv1 cell proliferation, measured by BrdU incorporation assay. Values are presented in fold change compared to control cells. Data represent means of three independent experiments and the bars represent SEM of the mean. * *p* < 0.05 ** *p* < 0.01 (**D**) Western blot analysis showing the expression levels of MCT2, PEX19, and TUB in control and MCT2-overexpressed and/or PEX19-silenced 22Rv1 cells. A densitometric quantification of the immunoblots is presented in Figure S1, since whole western blots are presented at Figure S5.

In order to analyze the impact of MCT2’s localization at peroxisomes on prostate cancer proliferation, we performed proliferation analyses using BrdU incorporation assays. Interestingly, the silencing of MCT2 resulted in a strong decrease in 22Rv1 cells proliferation (about 45%) compared to nonsilenced control cells (Figure 4A,B), indicating that MCT2 plays a critical role in PCa proliferation. To substantiate these results, we analyzed cell proliferation in 22Rv1 cells overexpressing Myc-MCT2. Our results demonstrate that MCT2 overexpression increases on cell proliferation (approximately 50%), compared to nontransfected cells (Figure 4C,D).

As most of the overexpressed Myc-MCT2 was targeted to peroxisomes in 22Rv1 cells (Figure 2B), we wondered whether the specific localization of MCT2 at this organelle would be important for its role on PCa cell proliferation. We hence assessed cell proliferation of 22Rv1 cells after the knockdown of the chaperone PEX19, inhibiting MCT2 trafficking to the peroxisomal membranes. Importantly, the overexpression of Myc-MCT2 in the absence of PEX19 did not induce the increase on cell proliferation observed in the presence of this chaperone (Figure 4C,D). These results clearly indicate that the specific localization of MCT2 at the peroxisomal membranes is essential for its role on 22Rv1 cells proliferation.

We performed the same experiments in PC3 cells (in which some of the MCT2 is also localized at peroxisomes) and obtained similar results (Figure S4). Altogether, these results suggest that the MCT2 shift to peroxisomes plays an important role in PCa cells proliferation.

## 3. Discussion

From malignant transformation to progression, PCa cells display a dynamic metabolism, which is remodeled according to the specific requirements of each tumor stage. We have previously suggested that malignant transformation is related to alterations in peroxisome morphology and to the presence of MCT2 at the organelle’s membranes, contributing to a redox shuttle system which supports β-oxidation and maintains redox balance [18]. In this study we further demonstrate that the metabolic switch along PCa initiation and progression is accompanied by the increase in the expression levels of key proteins involved in lipid metabolism. The increased expression of PMP70, ACBD5, CAT, PEX5, PEX19, PECI, ACOX1, ACOX3, SCPx, and ABCD1 in 22Rv1 cells, compared to nontumor cells, suggests that PCa take advantage of the peroxisomal lipid metabolism, upregulating the import of fatty acids (via PMP70, ABCD1, and ACBD5), and the classical peroxisome proliferator-inducible and noninducible pathways (via ACOX1 and ACOX3, respectively). These conclusions are substantiated by a significant increase of the peroxisomal β-oxidation, monitored by C26:0 oxidation, in 22RV1 cells when compared to nontumor cells. Our results furthermore indicate an increase in CAT expression, which may be directly related to an increased production of peroxisome-derived H_2_O_2_. Further analyses should be performed in order to determine whether reactive oxygen specieslevels are altered in these cells, as well as in the import of matrix and membrane peroxisomal proteins (via PEX5 and PEX19, respectively), to ensure higher peroxisomal metabolic capacity.

In later tumor stages, here represented by PC3 cells, the expression levels of peroxisomal PMP70, ACBD5, CAT, PEX5, and PEX19 decrease if compared to 22Rv1 cells, suggesting that their respective pathways may become less relevant with disease progression, in accordance with the switch to the Warburg effect that is observed at this stage [34]. Intriguingly, peroxisomal β-oxidation activities as well as the expression levels of ACOX1 and PECI are increased in PC3 cells when compared to 22Rv1 cells, suggesting that, although late stages rely on the Warburg effect for energy production, distinct peroxisomal lipid metabolic pathways are still relevant at these stages. In line with these findings, an antiproliferative effect was recently published after knockdown of PECI in LNCaP cells (another metastatic PCa cell line) [35]. Our results suggest that PCa remodels peroxisome metabolism according to the specific needs mainly at the initial but also later tumor stages. The very specific peroxisomal metabolism, which is not primarily associated with energy production, is indeed different between nontumor and tumor cells. This may point to a possibly more specific function of peroxisomes, e.g., in membrane lipid homeostasis changes during disease progression. These results may have uncovered a new aspect in disease pathology, which should certainly be followed-up in the future.

As the androgen response plays a crucial role in PCa [12,36], it would certainly be important to explore a possible relation between peroxisome metabolism and androgen signaling in these cells and during cancer progression in in vivo studies.

Although we did not assess the expression levels of mitochondrial β-oxidation enzymes, the increased expression of CPT1 in 22Rv1 cells suggests stimulation of the transport of acyl-CoAs to mitochondria. Furthermore, mitochondrial CPT1, COXIV, and ATP5A/B expression levels decrease in PC3 cells, suggesting a lesser relevance of the respective pathways for disease progression, in line with the observed changes in peroxisome metabolism. In accordance to these observations, a decrease in mitochondrial TCA cycle enzymes and a change in mitochondrial morphology were recently reported, when androgen-sensitive and androgen-insensitive tumor cell lines were compared by proteomic profiling [37]. Further corroborating our results, the authors could not detect changes in general mitochondrial mass and many not metabolism-associated mitochondrial proteins. The deregulation of apoptosis and metabolism in many cancers have been associated with upregulation of VDAC1 [38]. Our results showed an increase of VDAC1 expression in PC3 cells. In fact, the association of VDAC1 with the glycolytic enzymes hexokinases is associated with the rapid growth of malignant cells, increased glucose metabolism, and inhibition of apoptosis [39,40].

Peroxisome morphology in 22Rv1 cells is quite unique per se, as peroxisomes appear elongated and organized in clusters, when compared with most cells, including nontumor prostate cells, where these organelles assume a more spherical/rod morphology and individual organization [18]. Here we showed that this unusual morphology is exacerbated upon MCT2 overexpression, which induces an increase of peroxisomal surface and a decrease in peroxisome number. It is tempting to suggest that MCT2’s endogenous presence at this organelle might induce the elongated and clustered morphologies that are characteristic of 22Rv1 cells’ peroxisomes. It is, however, possible that this uncommon morphology is a result of the seemingly intensified peroxisome metabolism present in these cells. This would likely account for the fact that MCT2 knockdown did not reverse the organelle’s morphology in 22Rv1 cells. The peroxisome morphology in these cells may furthermore be a consequence of an imbalance in the membrane lipid/protein ratio. Our results also show that MCT2 overexpression in 22Rv1 cells did not affect the expression of key proteins involved in peroxisomal β-oxidation, which are already altered in comparison to nontumor cells.

Our results further demonstrate that MCT2 overexpression leads to an increase of 22Rv1 and PC3 cell proliferation, while MCT2 knockdown decreased the capability of the cells to proliferate. These results indicate that MCT2 is associated with PCa proliferation and, more specifically, that the peroxisomal localization of MCT2 seems to be required for PCa proliferation as, upon inhibition peroxisome membrane protein by PEX19 knockdown, MCT2 overexpression was not able to elevate the cells’ proliferative capacity. It would be important to, in future studies, manipulate the expression of different enzymes involved in the peroxisomal fatty acid oxidation, in order to unravel the importance of this metabolic pathway for PCa cells proliferation.

Although additional functional and in vivo analyses should be performed to further substantiate our results, altogether our data suggests a metabolically important role of peroxisomes in the initial, and to a lesser extend also later, PCa stages. It furthermore demonstrates the importance of peroxisomal MCT2 for localized tumor cells proliferation. Taken together, our results hence highlight the importance of peroxisomes for PCa development and expose a range of possible targets for PCa therapy.

## 4. Materials and Methods

### 4.1. Plasmids and Antibodies

In these experiments, the following antibodies were used: MCT2 (sc-50322; Santa Cruz Biotechnology, Santa Cruz, Dallas, TX, USA), PEX14 (a gift from Dr. Dennis Crane, Griffith University, Brisbane, Australia), CAT (ab88650; Abcam, Cambridge, UK), PMP70 (SAB4200181; Sigma-Aldrich, St. Louis, MO, USA), ACOX1 (a gift from A. Völkl, University of Heidelberg, Heidelberg, Germany), ACOX3 (HPA035840, Sigma-Aldrich, St. Louis, MO, USA), PEX19 (SAB1401323, Sigma-Aldrich, St. Louis, MO, USA), α-TUB (T9026, Sigma-Aldrich, St. Louis, MO, USA), α/β-TUB (2148, Cell signalling Technology, Beverly, MA, USA), PEX5 (a gift from Dr. Jorge Azevedo, University of Porto, Porto, Portugal), CPT1 (sc-514555, Santa Cruz Biotechnology, Santa Cruz, Dallas, TX, USA), BrdU (M0744, Dako, Glostrup, Denmark), TOM20 (612278, (BD Bioscience, San Jose, CA, USA), COXIV (ab33985, Abcam, Cambridge, UK), VDAC1 (ab15895, Abcam, Cambridge, UK), ACBD5 (HPA012145, Sigma-Aldrich, St. Louis, MO, USA), ACSL4 (sc-365478, Santa Cruz Biotechnology, Santa Cruz, Dallas, TX, USA), PECI (611822, BD Bioscience, San Jose, CA, USA), ACAA1 (a gift from A. Völkl, University of Heidelberg, Heidelberg, Germany), ATP5A (612516, BD Bioscience, San Jose, CA, USA), ATP5B (ab5432, Abcam, Cambridge, UK), TRITC (Jackson ImmunoResearch, West Grove, PA, USA), Alexa 488 (Invitrogen, Life Technologies, Carlsbad, CA, USA), HRP (BioRad, Hercules, CA, USA), ABCD1 (11159-1-AP, Proteintech, Manchester, UK), SCP2 (MBS1495100, San Diego, CA, USA), GAPDH abcam (ab8245, Abcam, Cambridge, UK), Myc (2278, Cell signalling Technology, Beverly, MA, USA), IRDye 800CW and IRDye 680RD (LI-COR Biotechonology, Cambridge, UK). The following primer sequences, including sites for *BamHI* and *XhoI*, were used to amplify the coding sequences MCT2, from human 22Rv1 cells: 5’ CGGGATCCAATGCCACCAATGCCAAGTG 3’ (forward) and 5’ CCGCTCGAGTTAAATGTTAGTTTCTCTTTCTG 3’ (reverse). To obtain an N-terminal Myc-tagged MCT2 construct, *BamHI* and *XhoI* were used to clone the cDNA in pCMV-Tag 3a vector (Agilent Technologies, La Jolla, CA, USA). The clone was verified by DNA sequencing.

### 4.2. Cell Culture and Transfection

PNT1A (nontumor SV40-immortalized prostate epithelial cells, no androgen expression), 22Rv1 (derived from localized prostate tumor, androgen-sensitive) and PC3 cell lines (derived from bone metastasis of prostate cancer, androgen-insensitive) (provided by Dr. Fátima Baltazar, University of Minho, Portugal) were seeded in RPMI-1640 (Gibco, Invitrogen, Carlsbad, CA, USA) supplemented with 10% of fetal bovine serum (Gibco, Invitrogen, Carlsbad, CA, USA), 1% of antibiotic (penicillin/streptomycin) (Gibco, Invitrogen, Carlsbad, CA, USA), and incubated at 37°C in an atmosphere containing 5% CO_2_. All cell lines were cultivated under the same experimental conditions and observations were made at about 70% cell confluence.

For overexpression analyses, 22Rv1 and PC3 cells were transfected with Myc-MCT2 using Lipofectamine 3000 (Invitrogen, Waltham, MA, USA) and collected after 24 h. MCT2 and PEX19 knockdown in 22Rv1 and PC3 cells was performed using Lipofectamine RNAiMAX (Invitrogen, Waltham, MA, USA). Two different siRNA, S17574 and S17572 (Ambion, Inc, Austin, TX, USA) were incorporated at final concentration of 50 nM to MCT2 knockdown. For PEX19 knockdown, S11612 at final concentration of 10 nM was used (Ambion, Inc, Austin, TX, USA). The transfections were performed according to the manufacturer’s instructions and cells were collected after 48 h.

### 4.3. Immunofluorescence and Microscopy Analyses

Immunofluorescence analyses were performed by seeding cells on glass coverslips that were fixed with 4% paraformaldehyde in PBS, pH 7.4 for 20 min. Afterwards, cells were permeabilized with 0.2% Triton X-100 for 10 min, blocked with 1% BSA solution for 10 min, and incubated with primary and secondary antibodies for 1h each. Between each step, cells were washed three times with PBS, pH 7.4. Lastly, cells were stained with Hoechst 33258 (PolySciences, Warrington, FL, USA) for nuclei staining and mounted in slides using Mowiol 4-88 containing n-propylgallate. Images were obtained using a Zeiss LSM 510 Meta Confocal setup (Carl Zeiss, Jena, Germany) equipped with a plan-Apochromat 100×/1.4 oil objective. Image analyses for peroxisome number and surface quantifications were obtained through Spot detector plugin from Icy software [41]. Peroxisome morphology quantifications were performed in at least 50 cells from three independent experiments. Quantification of colocalization was performed by determining the Pearson’s correlation coefficient using the JACoP software (version 2.0.0.0) [42].

For electron microscopy analyses cells were seeded on glass cover slips and fixed in 4% formaldehyde, 0.05% glutaraldehyde, 2% sucrose in PBS, pH 7.4 for 1h, at room temperature. Following a PBS pH 7.4 rinse, cells were further fixed with 1.5% glutaraldehyde in PBS pH 7.4 for 30 min, at room temperature. After it was performed the DAB staining, following Bonekamp et al. [43] and cells were post-fixed in 1% osmium in PBS for 1h on ice and in the dark and then en-bloc stained with 2% uranyl acetate in distilled water for 30 min on ice and in the dark. Cells were finally taken through a graded ethanol dehydration, embedded in EPON resin and polymerized at 60 °C, overnight. Sections of 70 nm were cut on a UC7 Ultramicrotome (Leica, Wetzlar, Germany), picked up on formvar coated slot grids and post-stained with uranyl acetate for 5 min and lead citrate for 5 min. Sections were imaged in a H7650 transmission electron microscope (Hitachi, Tokyo, Japan) operated at 100keV and images were recorded on XR41M mid mount AMT digital camera.

### 4.4. Immunoblotting

Cells were lysed with specific lysis buffer (25 mM Tris-HCl, pH 8.0, 50 mM sodium chloride, 0.5% sodium deoxycholate, 0.5% Triton X-100 and a protease-inhibitor mix). To improve protein extraction, samples were passed 20 times through a 26-gauge syringe needle and incubated on a rotary mixer at for 30 min at 4 °C. After cleared by centrifugation (17,000× *g*, 15 min), protein concentrations were determined by Bradford assay (Bio-Rad, Hercules, CA, USA). Blots were incubated with the specific primary and secondary antibodies and detected by HRP using an enhanced chemiluminescence system (GE Healthcare, Waukesha, WI, USA) or by Odyssey CLx (LI-COR Biotechonology, Cambridge, UK).

### 4.5. β-Oxidation Measurements

The β-oxidation rates of cerotic acid (C26:0) were measured in cultured cells using radioactive labeled C26:0 as previously described [44].

### 4.6. Cell Proliferation Assay

To evaluate the role of MCT2 in the proliferation of PCa, 22Rv1 and PC3 cells were seeded on glass cover slips for 24 h prior to MCT2 transfection or knockdown, and cell proliferation was assessed by BrdU incorporation assay. Upon 24 h of transfection, cells were incubated with 10µM BrdU (10280879001, Sigma-Aldrich, St. Louis, CA, USA) for 1 h at 37 °C, 5% CO_2_. After incubation, cells were fixed with 4% paraformaldehyde for 30 min and incubated in 2M HCl for 20 min at room temperature, for DNA denaturation. Cells were then incubated with primary and secondary antibodies for 30 min, stained with Hoechst 33258 and mounted in slides using Mowiol 4-88 containing n-propylgallate. Between each step before denaturation, cells were washed three times with PBS and after denaturation cells were washed with PBS-T-B (PBS, supplemented with 0.5% Tween 20 and 0.05% bovine serum albumin), pH 7.4. The BrdU-positive cells were counted under the confocal microscope.

### 4.7. Statistical Analyses

Statistical analysis was performed in Graph Pad Prism 5 (GraphPad Software, Inc., La Jolla, CA, USA), using Student’s *t*-test for comparison between two groups and one-way ANOVA, followed by Bonferroni’s for multiple comparison; data are presented as mean ± standard error mean (SEM). *p*-values of ≤0.05 were considered as significant.

## 5. Conclusions

With this work, we provided evidence that peroxisome metabolism plays an important role in PCa. Our results suggest that PCa takes advantage of the peroxisomal lipid metabolism, upregulating e.g., the peroxisomal transport of fatty acids, the classical peroxisome proliferator-inducible and noninducible pathways, ROS metabolism, and the import of matrix and membrane peroxisomal proteins, to ensure higher peroxisome metabolic capacity.

Importantly, we demonstrated that the localization of MCT2 at peroxisomes, which we suggest to induce alterations in the organelle’s morphology, is required for PCa proliferation.

Our results underline the role of peroxisomes in PCa development and uncover different cellular mechanisms that may be further explored as bases for novel PCa therapies.

## Supplementary Materials

The following are available online at https://www.mdpi.com/2072-6694/12/11/3152/s1, Figure S1: Western blot quantification of the expression levels of the different proteins highlighted in Figure 1, Figure 2, Figure 3, Figure 4, Figures S2, S3 and S4; Figure S2: Analysis of modifications in peroxisome morphology and number in PC3 cells transfected with Myc-MCT2, when compared with nontransfected cells; Figure S3: Analysis of modifications in peroxisome morphology and number in PC3 cells upon silencing of MCT2, when compared with nonsilenced cells. Figure S4: MCT2 localization at the peroxisomal membranes is associated with PCa proliferation in PC3 cells; Figure S5. Complete western blots used in (A) Figure 1, (B) Figure 2, (C) Figure 3, (D) Figure 4, Supplementary Figure S2, (F) Supplementary Figure S3 and (G) Supplementary Figure S4.
