# Supplementary material for: Prostate Cancer Proliferation Is Affected by the Subcellular Localization of MCT2 and Accompanied by Significant Peroxisomal Alterations
Source: Cancers (Basel). 2020 Oct 27;12(11):3152. doi: 10.3390/cancers12113152 (PMC7693163; doi:10.3390/cancers12113152)
Supplement: Supplementary file 1 [file cancers-12-03152-s001.zip › cancers-954479-supplementary_final 2_inserted figures.docx]

**Supplementary Materials**


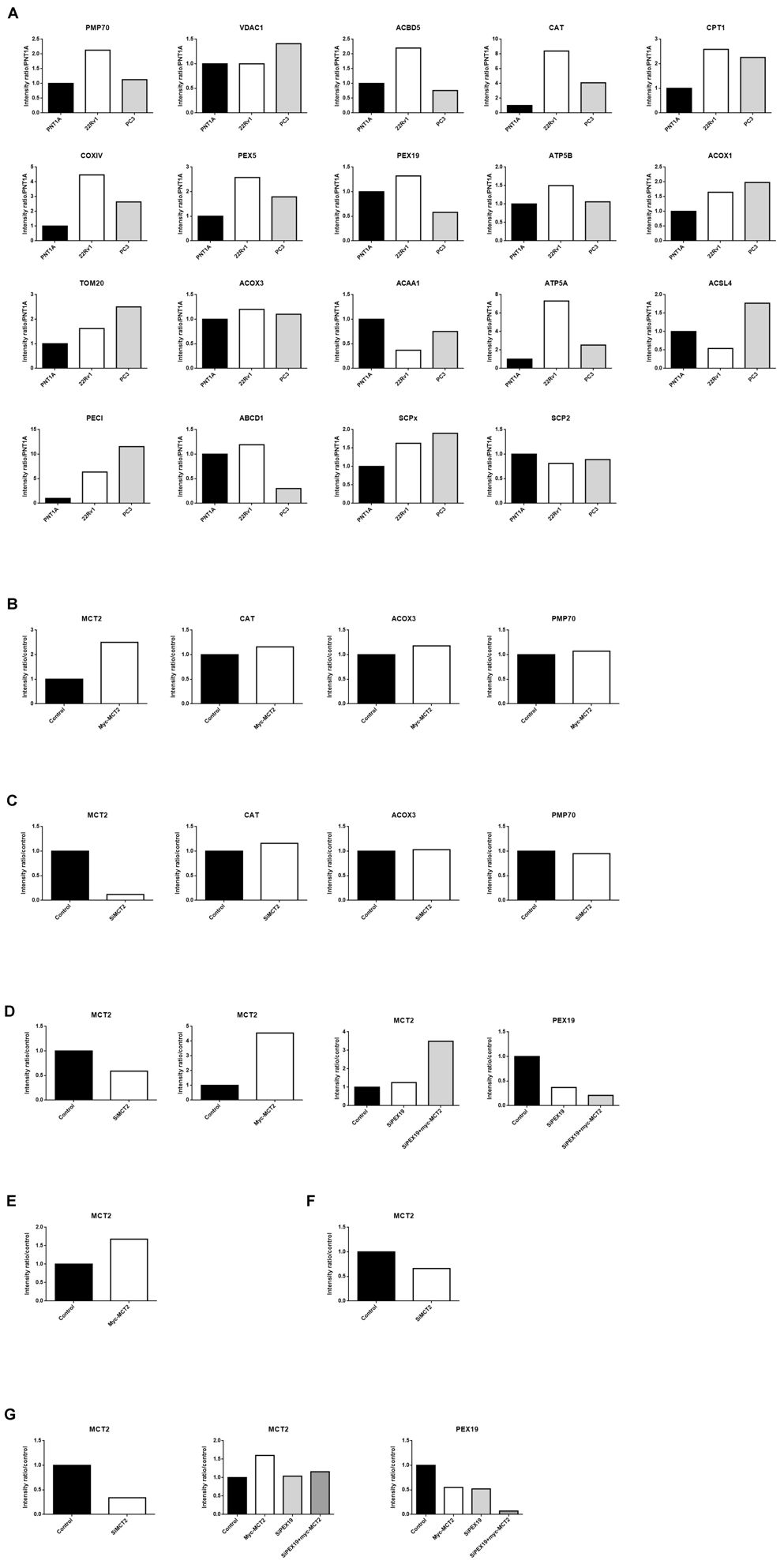


**Figure S1.** Western blot quantification of the expression levels of the different proteins highlighted in Figures 1, 2, 3, 4, S2, S3 and S4. (**A**) Western blot quantification of the expression levels of PMP70, VDAC1, ACBD5, CAT, CPT1, COXIV, PEX5, PEX19, ATP5B, ACOX1, TOM20, ACOX3, ACAA1, ATP5A, ACSL4, PECI, ABCD1 and SCP2, shown in Figure 1, in 22rv1 and PC3 cells, comparatively to PNT1A cells; (**B**) Western blot quantification of the expression levels of MCT2, CAT, ACOX3 and PMP70 in transfected cells, comparatively to control cells, shown in Figure 2; (**C**) Western blot quantification of the expression levels of MCT2, CAT, ACOX3 and PMP70 in MCT2 knockdown cells, comparatively to control cells, shown in Figure 3; (**D**) Western blot quantification of the expression levels of MCT2 and PEX19 in MCT2 knockdown and MCT2 overexpression in 22RV1 cells, before and after the knockdown of PEX19, comparatively to control cells, shown in Figure 4; (**E**) Western blot quantification of the expression levels of MCT2 in transfected cells, comparatively to control cells, shown in Figure S2, (**F**) Western blot quantification of the expression levels of MCT2 in MCT2 knockdown cells, comparatively to control cells, shown in Figure S3, (**G**) Western blot quantification of the expression levels of MCT2 and PEX19 in MCT2 knockdown and MCT2 overexpression in PC3 cells, before and after the knockdown of PEX19, comparatively to control cells, shown in Supplementary Figure S4. Tubulin and GAPDH were used as loading controls.


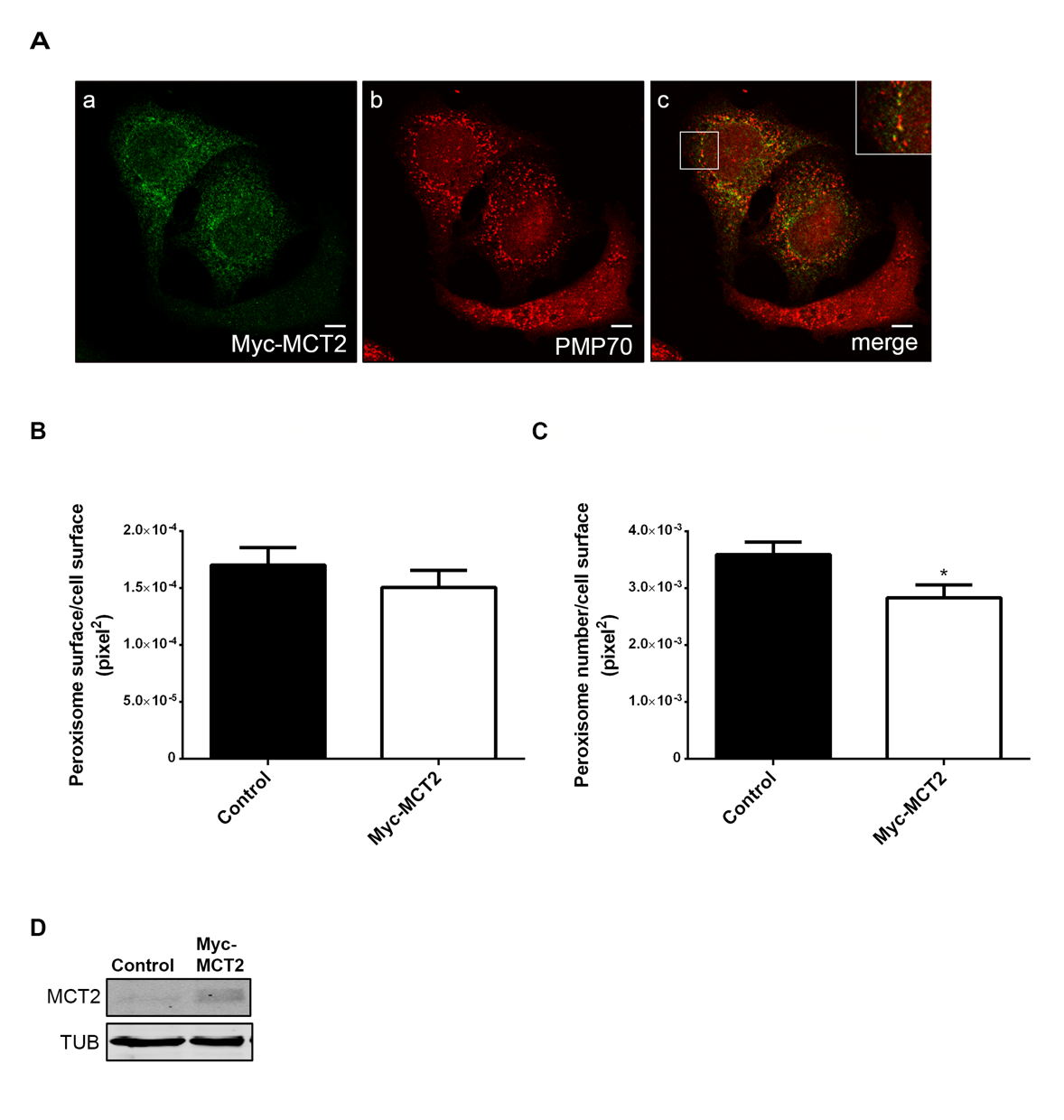


**Figure S2.** Analysis of modifications in peroxisome morphology and number in PC3 cells transfected with Myc-MCT2, when compared with nontransfected cells. (**A**) Immunofluorescence analysis of (a) Myc and (b) PMP70 by confocal microscopy in Myc-MCT2-transfected PC3 cells; (c) merge image of a and b; Bar represents 5 μm. (**B**,**C**) Analysis of modifications in peroxisome morphology and number in PC3 cells transfected with Myc-MCT2, when compared with not-transfected cells. Quantification analysis of alterations in peroxisomes’ area (pixel2), presented as the mean of peroxisome’ area per total cell area. (**D**) Western blot analysis showing the expression levels of MCT2 and TUB in control and Myc-MCT2-transfected PC3 cells. Data represent means of three independent experiments and the bars represented SEM of the mean. * *p* < 0.05.


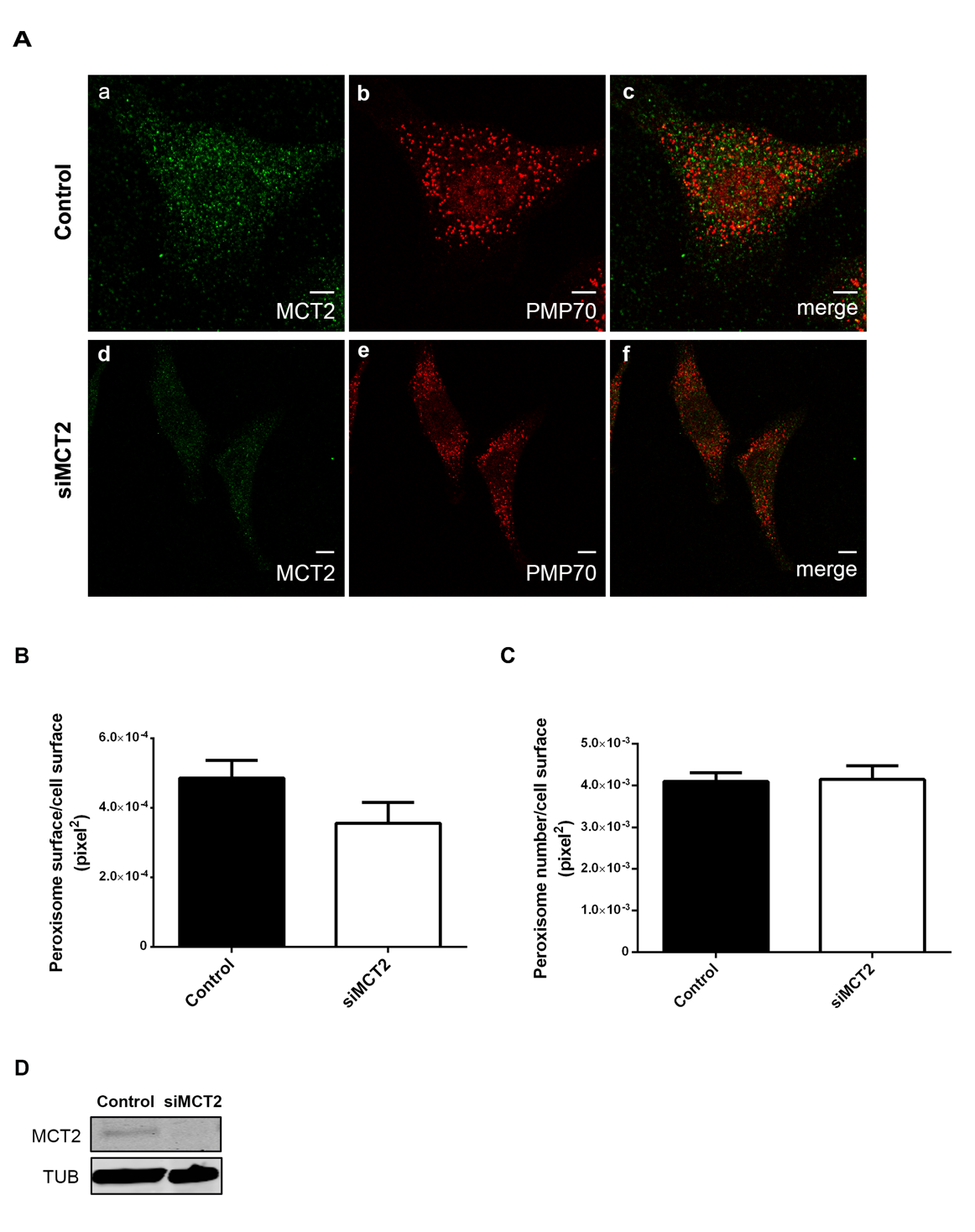


**Figure S3.** Analysis of modifications in peroxisome morphology and number in PC3 cells upon silencing of MCT2, when compared with nonsilenced cells. (**A**) Immunofluorescence analysis of (a,d) MCT2 and (b,e) PMP70 by confocal microscopy in PC3 cells; (c and f) merge image of a,b and d,e, respectively. Bars represent 5 μm. (**B**) Quantification analysis of alterations in peroxisomes’ area (pixel2), presented as the mean of peroxisome’ area per total cell area. (**C**) Quantification analysis of changes in peroxisomes’ number, presented as the mean of peroxisome number per cell area (pixel2). (**D**) Western blot analysis showing the expression levels of MCT2 and TUB in control and MCT2-silenced PC3 cells. Data represent means of three independent experiments and the bars represent SEM of the mean.


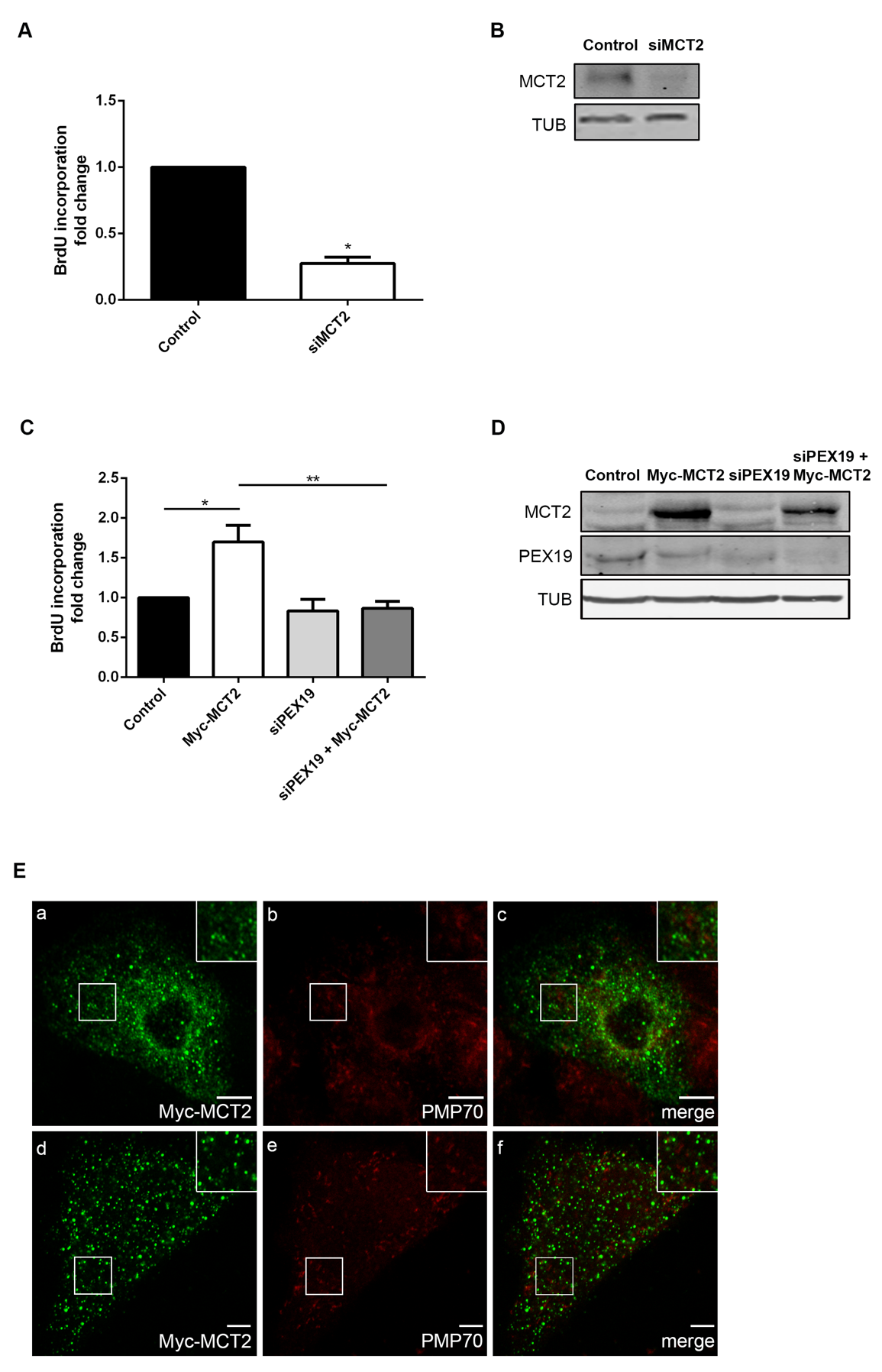


**Figure S4.** MCT2 localization at the peroxisomal membranes is associated with PCa proliferation in PC3 cells (**A**) Effect of the silencing of MCT2 on PC3 cell proliferation, measured by BrdU incorporation assay. Values are presented in fold change compared to control cells. Data represent means of three independent experiments and the bars represent SEM of the mean. * *p* < 0.05 ** *p* < 0.01 (**B**) Western blot analysis showing the expression levels of MCT2 and TUB in control and MCT2-silenced PC3 cells. (**C**) Effect of MCT2 overexpression, silencing of PEX19 and Myc-MCT2 overexpression in the absence of Pex19 on PC3 cell proliferation, measured by BrdU incorporation assay. Values are presented in fold change compared to control cells. Data represent means of three independent experiments and the bars represent SEM of the mean. * *p* < 0.05 ** *p* < 0.01 (**D**) Western blot analysis showing the expression levels of MCT2, PEX19 and TUB in control and MCT2-overexpressed and/or PEX19-silenced PC3 cells. Data were the means of three independent experiments and the bars represented SEM of the mean. * *p* < 0.05 ** *p* < 0.01. A densitometric quantification of the immunoblots is presented in Figure S1. (**E**) Immunofluorescence analysis of (a,d) Myc-MCT2 and (b,e) PMP70 by confocal microscopy in Myc-MCT2-transfected 22Rv1 (a–c) and PC3 (d–e) cells in the absence of Pex19; (c,f) merge image of a and b. Bars represent 5 μm.


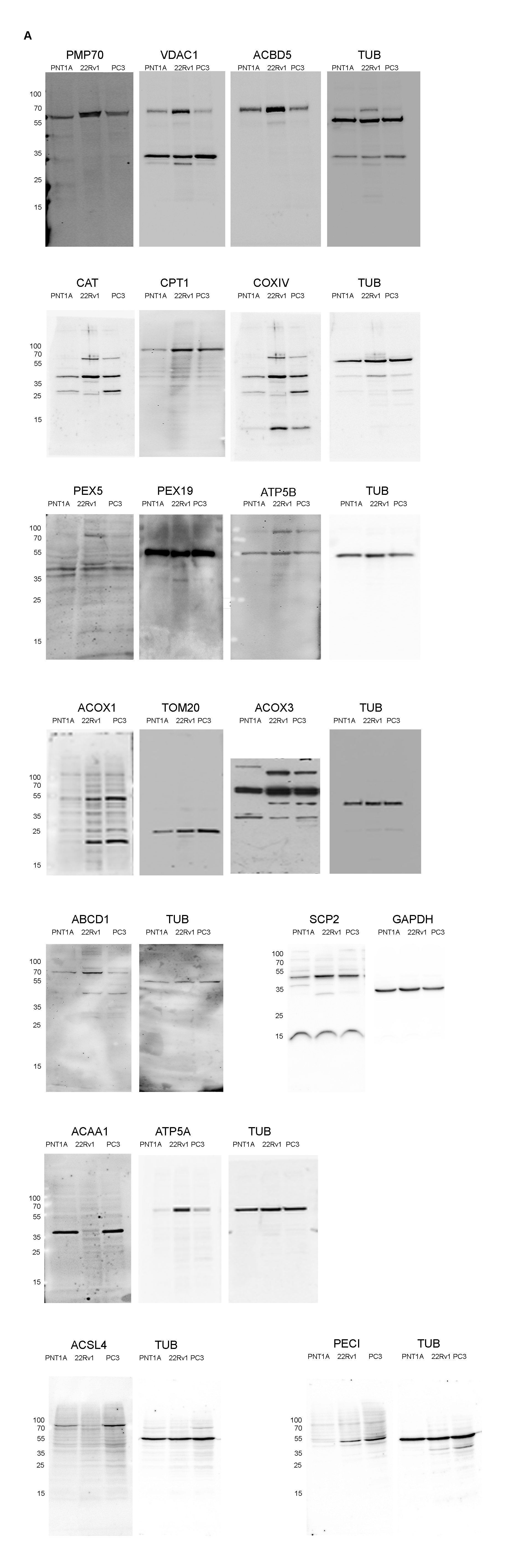


**Figure S5.** *Cont.*


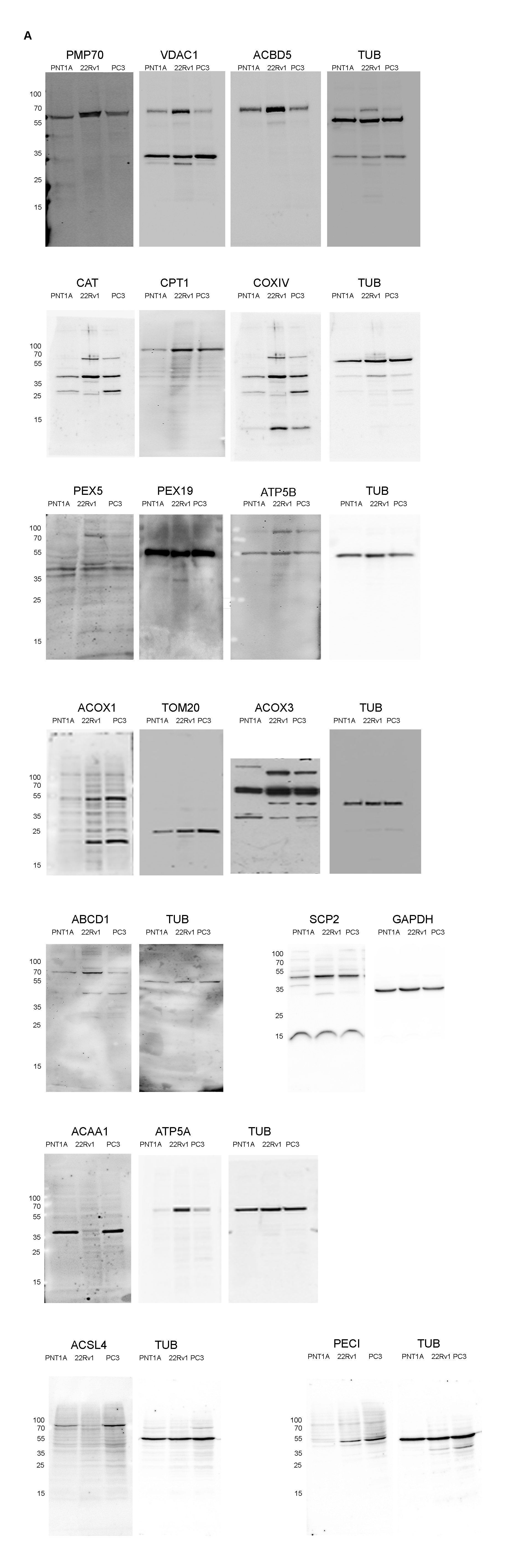


**Figure S5.** *Cont.*


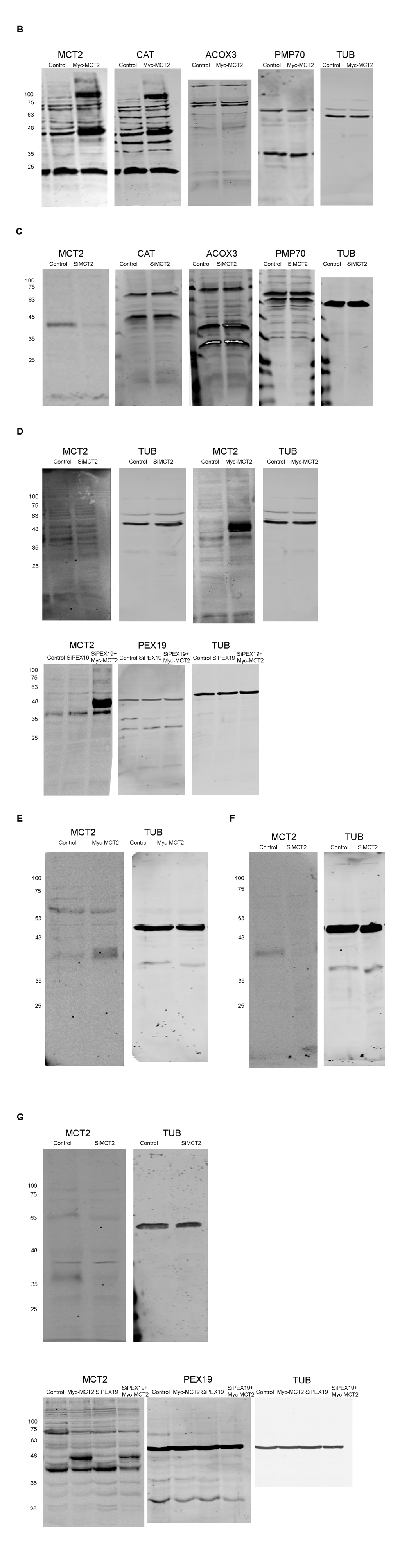


**Figure S5.** *Cont.*


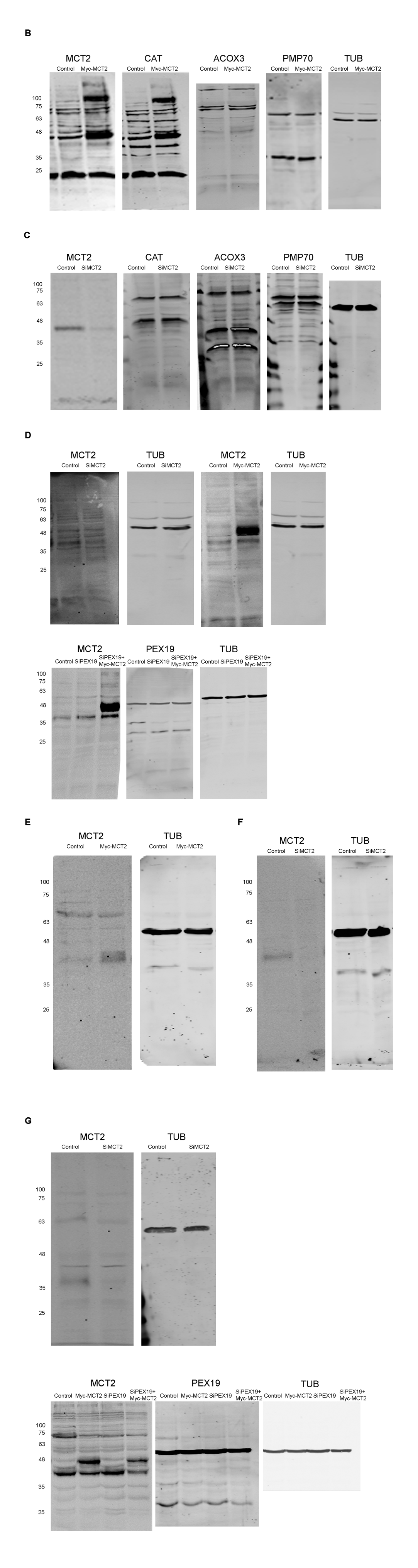


**Figure S5.** Complete western blots used in (**A**) Figure 1, (**B**) Figure 2, (**C**) Figure 3, (**D**) Figure 4, Supplementary Figure 2, (E,**F**) Supplementary Figure 3 and (**G**) Supplementary Figure S4.
